# Impact of Glucocorticoid Replacement Therapy on Nocturnal Hypoglycemia in Adrenal Insufficiency: An Analysis of Multiple Case Studies

**DOI:** 10.7759/cureus.61456

**Published:** 2024-05-31

**Authors:** Ken Kanazawa, Mai Hijikata, Shinichiro Koga, Koichiro Kuwabara

**Affiliations:** 1 Department of Diabetes, Metabolism, and Endocrinology, Japan Labor Health and Safety Organization, Tokyo Rosai Hospital, Tokyo, JPN

**Keywords:** continuous glucose monitoring, quality of life, glucocorticoids, nocturnal hypoglycemia, adrenal insufficiency

## Abstract

Background and aim: Adrenal insufficiency (AI) is a hormonal disorder characterized by insufficient glucocorticoid production. Nocturnal hypoglycemia (NH) occurs in patients with AI. However, the effect of glucocorticoid replacement therapy (GCRT) on AI and NH remains unclear. This study aimed to investigate the relationship between AI and NH by evaluating the impact of GCRT on NH in patients newly diagnosed with AI.

Methods: The present study was conducted between October 2018 and December 2022 at the Department of Diabetes, Metabolism and Endocrinology of the Tokyo Rosai Hospital, Japan. In total, 15 patients aged ≥18 years with newly diagnosed AI or NH were included in this study. The NH frequency was measured using continuous glucose monitoring (CGM). The primary outcome was the change in NH frequency before and after the GCRT intervention.

Results: GCRT significantly decreased NH frequency. Severe NH frequency and minimum nocturnal glucose levels changed significantly while fasting blood glucose and glycated hemoglobin levels did not change significantly. GCRT intervention improved CGM profiles’ time below range, time in range, and average daily risk range.

Conclusions: The present study suggests that GCRT can help newly diagnosed patients with AI manage NH. These findings show that CGM can detect NH in patients with newly diagnosed AI, determine the optimal GCRT dosage, and hence prevent an impaired quality of life and even serious adverse effects in these patients. Further large multicenter studies should validate these findings and delve deeper into the mechanistic link between AI and NH.

## Introduction

Adrenal insufficiency (AI) is an endocrine disorder characterized by absolute or relative production of glucocorticoids [1-5]. This condition is typically accompanied by fatigue, reduced energy and appetite, hypotension, and weight loss. Notably, recent reports have highlighted the occurrence of nocturnal hypoglycemia (NH) in patients with AI [6-9]. The advent of continuous glucose monitoring (CGM) devices has allowed the detection of NH in these patients, thereby helping prevent significant impairment in their quality of life and serious adverse effects [6,8]. NH can also manifest in individuals without chronic diseases, such as diabetes which may result from various factors, including skipping meals, excessive alcohol consumption, advanced chronic kidney disease, and reactive hypoglycemia [10-12]. In association with AI and NH, cortisol deficiency may cause hypoglycemia due to decreased glucose production in the liver and decreased glucose uptake in peripheral tissues. The effect of glucocorticoid replacement therapy (GCRT) on AI and NH remains unclear. This study aimed to investigate the relationship between AI and NH by evaluating the impact of GCRT on NH in patients newly diagnosed with AI. This study is unique because it focused on a specific population of newly diagnosed AI patients with NH, which has not been reported previously. The findings of this study hold the promise of providing critical insights into the management of AI and NH, ultimately improving the patient's quality of life.

This article was previously published as a preprint in Research Square on October 3, 2023.

## Materials and methods

Study design and ethical considerations

The present study was conducted at the Department of Diabetes, Metabolism and Endocrinology, Tokyo Rosai Hospital, Japan, between October 2018 and December 2022. Participants provided written or oral informed consent and were allowed to opt out of the study. Eligibility assessments were conducted for all participants. Two endocrinologists reviewed data in accordance with the Declaration of Helsinki. The study protocol was approved by the Institutional Review Board of Tokyo Rosai Hospital (REC No.05-03).

Study participants

We assessed patients aged ≥18 years with newly diagnosed AI who presented with NH using CGM. NH was defined as a glucose level <70 mg/dL between 0 a.m. and 6 a.m. [13], measured using a Freestyle Libre Pro monitor (Abbott, Tokyo, Japan) [14,15]. Patients wore the monitor for 14 days, and data from days 2 to 14 were analyzed for NH events between 0 a.m. and 6 a.m. AI was suspected with clinical symptoms or laboratory findings suggestive of the disease, such as general malaise, hypotension, weight loss, eosinophilia, and electrolyte abnormalities, and those with basal cortisol levels <18 µg/dL (6-8 a.m.). A definitive diagnosis was made. Endocrinological stress tests were performed on admission. Peak cortisol levels <18 µg/dL following a standard-dose corticotropin (SDST) stimulation test or <20 µg/dL after a low-dose corticotropin stimulation test (LDST) were considered indicative of AI. Similarly, peak cortisol levels <18 µg/dL after a corticotropin-releasing hormone stimulation test (CRH) were diagnostic of AI [3,4]. Patients with myocardial infarction within three months prior to study enrollment, currently diagnosed with malignancy, acute infection, recent surgery or severe trauma, excessive alcohol consumption, anemia (hemoglobin <11 g/dL), severe liver disease, dialysis patients, and type 1 diabetes or diabetes using hypoglycemic drugs were excluded. In addition, we excluded patients with endogenous hyperinsulinism (insulinoma, functional beta-cell disorders, and autoimmune insulin hypoglycemia), hormone deficiency (adult growth hormone deficiency), and severe malnutrition as this study focused on chronic nonspecific symptoms of hormonal imbalance. We determined serum cortisol and plasma adrenocorticotropic hormone (ACTH) levels using an electrochemiluminescence immunoassay on the Cobas-8000 system (Roche Diagnostics, Basel, Switzerland). The established reference ranges were 7.1-19.6 μg/dL for serum cortisol and 7.2-63.3 pg/mL for plasma ACTH.

Study intervention

The intervention used in this study was GC-RT. Hydrocortisone (HC) dosage was adjusted based on the severity of AI and individual patient factors, including body weight and comorbidities. However, the HC dose had to be titrated based on the patient's symptoms of cortisol deficiency and excess.

Study outcomes

The primary study outcome was the change in the NH frequency (%) before and after GCRT. The secondary study outcomes were as follows: (1) assessment of blood glucose profile - fasting blood glucose level (FPG, mg/dL) and glycated hemoglobin (HbA1c) (%); (2) laboratory findings - white blood cell (WBC) (10^2^/μL), eosinophil (%), hemoglobin (Hb) (g/dL), hematocrit (Ht) (%), alanine aminotransferase (ALT) (U/L), sodium (mEq/L), potassium (mEq/L), estimated glomerular filtration rate (eGFR) (mL/min/1.73 m^2^), uric acid (UA) (mg/dL), high density lipoprotein-cholesterol (mg/dL), low density lipoprotein-cholesterol (mg/dL); (3) nocturnal glucose profile - frequency of severe NH (SNH) (glucose level ≤54 mg/dL from 0 a.m. to 6 a.m. {%}), minimum nocturnal glucose level (mg/dL), mean nocturnal glucose level (mg/dL), and assessed via CGM; (4) glycemic variability profile assessed via CGM - CGM time (times/13 days), time below range (TBR) (probability of reaching a glucose level below the treatment range {%}), time in range (TIR) (probability of reaching a particular blood glucose level 70-180 mg/dL {%}), time above range (probability of reaching a high-value range {%}), coefficient of variation (%CV) (0 a.m.-24 p.m. and 0 a.m.-6 a.m.), mean amplitude of glycemic excursions (MAGE) (0 a.m.-24 p.m. {%} and a.m.-6 a.m. {%}), mean of daily difference of blood glucose (MODD) (0 a.m.-24 p.m. {%} and 0 a.m.-6 a.m. {%}), and average daily risk range (ADRR) (0 a.m.-24 p.m. {%} and 0 a.m.-6 a.m. {%}), before and after the GCRT intervention.

Data collection and analysis

Categorical variables were expressed as raw frequencies and compared between the two groups using Pearson's chi-square test. Continuous variables with normal distribution were expressed as mean ± standard deviation and compared using Student's t-test. In contrast, continuous variables with non-normal distribution were expressed as median (interquartile range) and compared using the Wilcoxon signed-rank test. Analysis of covariance was used to compare changes in efficacy variables over time between the two groups. The significance level was set at p<0.05. All tests were two-tailed. Statistical analyses were performed using the JMP software version 12 (SAS Institute Inc., Cary, NC).

## Results

Study participants, clinical characteristics, and endocrinological assessment

**Figure 1:**
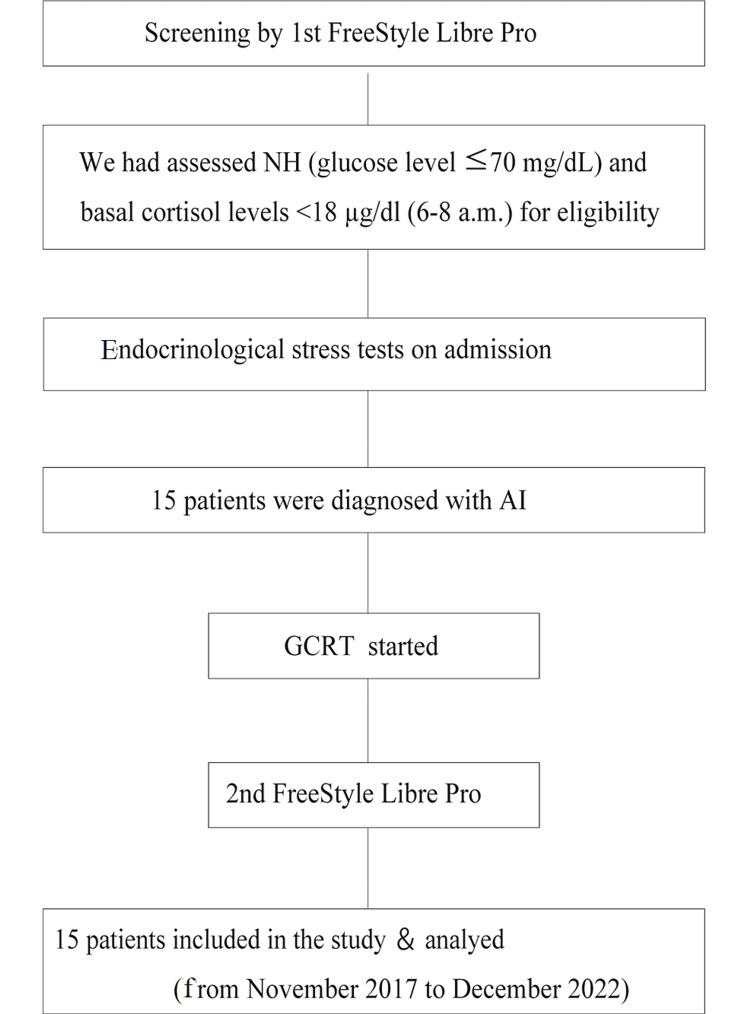
Flow diagram showing enrollment, study intervention, and follow-up of the study participants Freestyle Libre Pro monitor (Abbott, Tokyo, Japan). NH: nocturnal hypoglycemia; AI: adrenal insufficiency; GCRT: glucocorticoid replacement therapy

Fifteen patients with newly diagnosed AI who presented with NH were enrolled in this study (Figure 1). Table 1 shows the baseline clinical characteristics and results of endocrinological stimulation tests on admission. The mean basal cortisol level was 8.7±2.3 μg/dL, the mean basal ACTH level was 23.4±17.9 μg/dL, the peak cortisol level after LDST was 16.2±2.6 μg/dL, SDST was 20.3±3.9 μg/dL, and CRH was 14.7±2.8 μg/dL. In the endocrinological evaluation, two were newly diagnosed primary AI (PAI) (13.3%) and 14 were newly diagnosed secondary/tertiary AI (SAI/TAI) (93.3%). In the study intervention, the mean HC dosage was 6.0±2.3 mg/day.

**Table 1:** Comparison of the patients’ baseline characteristics Continuous data are expressed as mean±SD. GCRT: glucocorticoid replacement therapy; BMI: body mass index; DM: diabetes mellitus; OHA: oral hypoglycemic agent; eGFR: estimated glomerular filtration rate; BC: basal cortisol; PC: peak cortisol; LDST: low-dose corticotropin stimulation test; SDST: standard-dose corticotropin stimulation test; CRH: corticotropin-releasing hormone stimulation test; HC: hydrocortisone; F: female; M: male; CAI: central adrenal insufficiency; PAI: primary adrenal insufficiency; TAI: tertiary adrenal insufficiency; A/S: symptomatology; T2: type 2 diabetes mellitus; N/DM: no diabetes mellitus; N/A: no abnormality; SD: standard deviation

Case	Basal state									Endocrinological basal value						GCRT
	Age (years)	Sex	BMI (kg/m^2^)	Diagnosis	Symptoms	Alcohol use	DM	OHA use	eGFR (mL/min/1.73 m^2^)	BC (μg/dL)	ACTH (μg/dL)	PC (LDST, μg/dL)	PC (SDST, μg/dL)	PC (CRH, μg/dL)	Pituitary gland MRI	HC (mg/day)
1	78	M	23.4	CAI	A/S	No	T2	No	70.9	6.9	22.4	17.9	25.3	12.4	N/A	5
2	73	M	19.4	PAI	A/S	Yes	T2	No	23	9.2	69.2	15.1	17.1	12.1	N/A	7.5
3	79	M	22.7	CAI	A/S	Yes	T2	No	39.9	5.9	6.1	16.2	21.2	15.9	N/A	5
4	72	F	28.3	CAI	Nausea	No	T2	No	47.7	8	14.7	17.2	23.1	15.3	Empty cell	5
5	86	F	22.0	CAI	Fatigue	No	N/DM	No	44.3	10.9	11.4	17.7	20.3	14.6	Ratke cyst	5
6	75	M	18.6	CAI	A/S	No	T2	No	50.1	9.5	48.5	17.7	20.7	16.4	N/A	5
7	72	F	19.3	CAI	Nausea	No	T2	No	26.2	6.8	18.7	11.5	16.1	7.2	N/A	10
8	76	F	26.7	CAI	A/S	No	T2	No	56.7	7.9	10.3	17.1	23	15.4	N/A	5
9	28	F	16.6	TAI	Body weight loss	No	N/DM	No	78.9	7.8	11.5	14.1	17.3	15.9	N/A	12.5
10	46	F	23.9	CAI	Fatigue	No	N/DM	No	62.2	6.9	24.5	11	11	12.6	N/A	5
11	76	M	24.9	CAI	Dizziness	Yes	N/DM	No	63.8	14.4	22.7	19.1	25.3	14.6	Pituitary cyst	5
12	36	F	21.1	CAI	Fatigue	No	N/DM	No	76.9	6.3	48.6	14.3	18.1	15.8	N/A	5
13	53	F	21.5	PAI +CAI	A/S	Yes	N/DM	No	48.5	12	12.4	18.1	19.7	16.2	N/A	5
14	51	F	19	CAI	Fatigue	No	N/DM	No	60.92	9.4	15.9	16.8	21.7	19.2	N/A	5
15	48	F	19.7	CAI	A/S	No	N/DM	No	62.5	8.4	13.4	19.8	23.9	16.6	N/A	5
Mean ± SD	63.3 ±17.9	-	21.8 ±3.2	-	-	-	-	-	54.2 ±16.5	8.7 ±2.3	23.4 ±17.9	16.2 ±2.6	20.3 ±3.9	14.7 ±2.8	-	6.0 ±2.3

Change in the frequency of NH before and after the GCRT intervention

**Figure 2:**
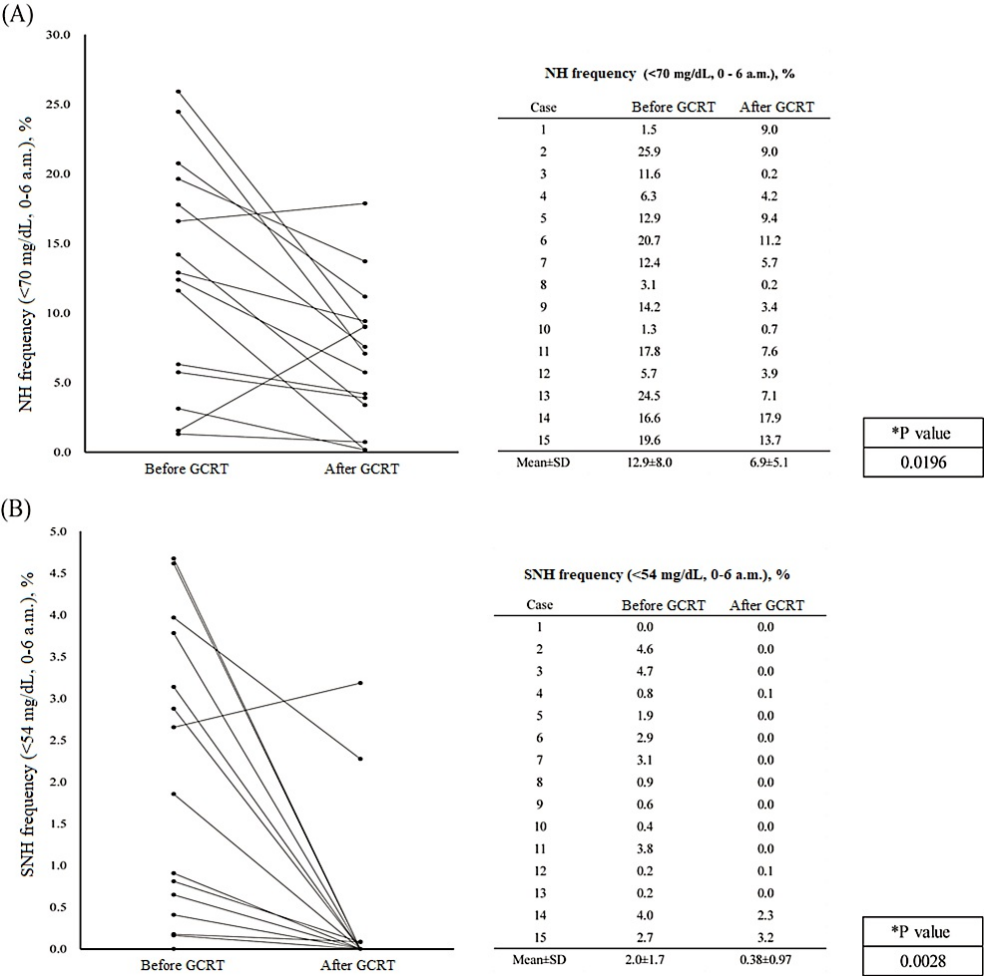
Change in frequency of nocturnal hypoglycemia and severe nocturnal hypoglycemia pre- and post-glucocorticoid replacement therapy (a) The frequency of NH (<70 mg/dL, 0–6 a.m.) before and after GCRT. (b) SNH frequency (<54 mg/dL, 0–6 a.m.) before and after GCRT. *Comparison of NH or SNH frequencies before and after GCRT using Student's t-test. NH: nocturnal hypoglycemia; SNH: severe nocturnal hypoglycemia; GCRT: glucocorticoid replacement therapy

The frequency of NH (%) significantly decreased after the GCRT intervention (mean NH events before GCRT: 12.9±8.0%; mean NH events after GCRT: 6.9±5.1%; p=0.0196) (Figure 2). Regarding the blood glucose profile, measures, such as FPG (mg/dL) and HbA1c (%), did not change significantly lower after the GCRT intervention (76.0±10.2 mg/dL vs. 82.2±12.9 mg/dL; p=0.158, 5.0±0.6 % vs 5.1±0.5 %; p=0.50) (Table 2). Nocturnal glucose profile: frequency of SNH (%) and minimum nocturnal glucose level (mg/dL) significantly changed (2.0±1.7% vs 0.38±0.97%; p=0.0028, 45.0±6.4 mg/dL vs 55.7±7.4 mg/dL; p=0.0002) (Figure 2 and Table 2). As per CGM profiles, TBR (<70 mg/dL, 0 a.m.-24 p.m. {%}), TIR (0 a.m.-24 p.m. {%}), and ADRR (0 a.m.-6 a.m. {%}) significantly changed after GCRT (24.8{7.1-35.6} % vs. 8.1{4.3-11.9} %; p=0.03, 74.8{61.0-87.4} % vs. 89.9{84.5-94.9} %; p=0.002, 14.7±6.1 vs. 12.1±3.9; p=0.005).

**Table 2:** Blood glucose profiles, laboratory findings, nocturnal glucose profile, and CGM profiles between pre- and post-GCRT *Comparison of values between patients before and after GCRT using Pearson’s chi-square test for categorical variables, and Student’s t-test and Wilcoxon signed-rank test for normally distributed and skewed continuous variables. GCRT: glucocorticoid replacement therapy; CGM: continuous glucose monitoring; FPG: fasting blood glucose level; WBC: white blood cell; Hb: hemoglobin; Ht: hematocrit; ALT: alanine aminotransferase; eGFR: estimated glomerular filtration rate; UA: uric acid; HDL-C: high density lipoprotein-cholesterol; LDL-C: low density lipoprotein-cholesterol; TBR: time below range; TIR: time in range; TAR: time above range; %CV: coefficient of variation; MAGE: mean amplitude of glycemic excursions; MODD: mean of daily difference of blood glucose; ADRR: average daily risk range; NS: not significant

	Pre-GCRT (n=15)	Post-GCRT (n=15)	p-Value
Blood glucose profiles			
Fasting glucose, mg/dL	76.0±10.2	82.2±12.9	NS
Laboratory findings			
WBC, 10^2 ^μL	51.1±15.5	57.7±16.6	NS
Eosino, %	4.1±4.1	2.2±1.7	NS
Hb, g/dL	12.4±1.3	12.9±1.6	NS
Ht, %	37.0±3.8	38.4±4.01	NS
ALT, U/L	14.8±5.4	16.4±4.8	NS
Na, mEq/L	141.2±2.6	140.9±2.9	NS
K, mEq/L	4.2±0.3	4.3±0.3	NS
eGFR, mL/min/1.73m^2^	54.2±16.5	55.6±18.2	NS
UA, mg/dL	5.4±1.3	5.2±1.3	NS
HDL-C, mg/dL	62.4±21.6	71.1±22.1	NS
LDL-C, mg/dL	104.1±31.2	101.1±26.8	NS
Nocturnal glucose profile			
Minimum glucose level (0 a.m.-6 a.m.), mg/dL	45.0±6.4	55.7±7.4	0.0002
Mean glucose level (0 a.m.-6 a.m.), mg/dL	76.5±9.8	82.4±12.5	NS
CGM profiles			
Time, times/13 days	1222.9±34.6	1215.4±56.4	NS
TBR (<70 mg/dL, 0 a.m.-24 p.m.), %	24.8 (7.1-35.6)	8.1 (4.3-11.9)	0.03
TIR (70-180 mg/dL, 0 a.m.-24 p.m.), %	74.8 (61.0-87.4)	89.9 (84.5-94.9)	0.002
TAR (>181 mg/dL, 0 a.m.-24 p.m.), %	0.7 (0.3-1.4)	1.0 (0.5-2.8)	NS
%CV (0 a.m.-24 p.m.)	31.1±10.0	28.8±9.7	NS
%CV (0 a.m.-6 a.m.)	5.5±3.1	4.7±2.4	NS
MAGE (0 a.m.-24 p.m.), mg/dL	37.3±28.2	37.9±25.0	NS
MAGE(0 a.m.-6 a.m.), mg/dL	7.9±4.9	8.0±4.9	NS
MODD (0 a.m.-24 p.m.), mg/dL	12.7±2.9	12.1±6.0	NS
MODD (0 a.m.-6 a.m.), mg/dL	5.8±3.7	6.1±3.9	NS
ADRR (0 a.m.-24 p.m.), mg/dL	27.1±13.0	21.1±10.3	NS
ADRR (0 a.m.-6 a.m.), mg/dL	14.7±6.1	12.1±3.9	0.005

Adverse events

In this study, none of the patients developed “severe” hypoglycemia, which indicates cognitive impairment requiring external assistance for recovery [13], during the CGM period, and none developed serious adverse events related to NH. A few patients experienced mild adverse events associated with using the sensor device.

## Discussion

This study investigated the relationship between AI and NH by evaluating the effect of GCRT on NH in 15 patients newly diagnosed with AI. The results demonstrated a significant decrease in the frequency of NH following GCRT intervention, highlighting the potential role of HC in managing NH in patients with AI.

A new finding of our study was the significant reduction in NH and SNH frequencies following GCRT intervention in patients with AI. Additionally, it is interesting that a minimal HC dosage improved the quality of glucose variability throughout the day. AI therapy aims to rehabilitate patient health by administering appropriate amounts of glucocorticoids to mimic the physiological circadian secretion of serum cortisol [1,16-18]. However, it remains challenging to attain a physiological cortisol profile because current therapies cannot replicate the physiological circadian rhythm of cortisol, and premature mortality is still reported in patients with AI [19,20]. In determining the optimal dose of HC for an individual patient, physical findings, such as weight, health status, and blood pressure, among others, and the subjective well-being of the patient [3,4,21,22] are frequently taken into account as well as the lack of definitive indicators [1]. Increased chronic exposure to glucocorticoids has been recognized as a potential cause of deterioration in subjective health status, weight, and glucose metabolism in patients with AI [1,16-18,23]. However, when cortisol levels are lower, late-night and early-morning hypoglycemia may be more likely to be missed because patients are often unconscious [6-8]. A previous study demonstrated the effectiveness of an alternative approach to the management of patients with AI, in which the adequacy of HC dosage was assessed by monitoring glucose levels using CGM [6-8]. The findings of this study are consistent with those of several previous studies, suggesting the effectiveness of CGM in determining the optimal dose of HC in patients with AI. However, this study extends these findings by revealing that GCRT directly influences NH in patients newly diagnosed with AI, providing novel perspectives on the AI-NH relationship. In several patients with CAI with relatively preserved function, the introduction of a low dose of HC (5 mg once daily upon awakening) improved the NH profile and prevented glucocorticoid overdoses. Of course, in many cases, the hypoglycemic condition persisted, necessitating future escalation of the HC dosage or the introduction of levofloxacin. Within the context of clinical practice, it is necessary to evaluate the improvement in headache and fatigue associated with NH and rule out other potential causative factors.

Particular attention must be paid to NH in older patients with AI and their underlying medical conditions. Hypoglycemia is a common and frequent adverse event, especially in patients with diabetes who receive insulin therapy or insulin secretagogues, and may cause increased mortality [24,25]. Hypoglycemia can be nonspecific and difficult to recognize, especially NH, in which signs are not noticed when blood glucose levels drop and can quickly become severe, leading to coma [13,26]. The mean age in this study was 63.3 ± 17.9 years, with nine patients >60 years and a relatively large number of patients with poor renal function (eGFR = 54.2±16.5 mL/min/1.73 m^2^). NH in older patients increases in patients >60 years of age and is most common in patients >80 years of age. The progression of chronic renal failure enhances the effects of insulin and increases the certainty of NH [11,27]. However, even in CAI with relatively preserved function, we find the possibility that NH may appear depending on age and patient medical conditions. Notably, there was no insulin therapy or insulin secretagogue; however, AI may have been involved in the development of SNH. Furthermore, to avoid SNH, repeated hypoglycemia must be avoided regularly [25]. The significant reduction in the frequency of NH events after the GCRT intervention in this study is expected to prevent SNH.

Limitations

This study has several limitations. First, this study was conducted in a single-center diabetes endocrinology department. Thus, the results may not be generalizable. Second, we need to consider the inaccuracy of the Freestyle LibrePro data at low glucose levels. Particularly, on the first day of wearing the sensor, the difference between the value measured by the sensor and the actual blood glucose level might have been significant. Therefore, CGM data were extracted and limited to days 2-14. Finally, confounding factors affecting the NH were not included in this study.

## Conclusions

Despite its limitations, this study provided novel perspectives on the AI-NH relationship and demonstrated that GCRT directly influenced NH in newly diagnosed patients with AI. These findings suggested that CGM can detect NH in patients with newly diagnosed AI, determine the optimal GCRT dosage, and hence prevent an impaired quality of life and even serious adverse effects in these patients. Further, large multicenter studies are needed to confirm the results of this study and delve deeper into the mechanistic link between AI and NH. Such investigations will enhance our understanding of and facilitate the development of better therapeutic approaches for this population.
